# Hypoglycemic effect and mechanism of isoquercitrin as an inhibitor of dipeptidyl peptidase-4 in type 2 diabetic mice

**DOI:** 10.1039/c8ra00675j

**Published:** 2018-04-19

**Authors:** Lei Zhang, Shi-Tao Zhang, Yan-Chun Yin, Shu Xing, Wan-Nan Li, Xue-Qi Fu

**Affiliations:** Edmond H. Fischer Signal Transduction Laboratory, School of Life Sciences, Jilin University Changchun 130012 China; College of Life Science and Technology, Mudanjiang Normal University Mudanjiang 157011 China

## Abstract

Glucagon-like peptide (GLP)-1 is a potent glucose-dependent insulinotropic gut hormone released from intestinal L cells. The aim of this study was to investigate isoquercitrin as an inhibitor of dipeptidyl peptidase IV (DPP-IV) and determine whether it affects GLP-1 release in normal mice and NCI-H716 cells. *In vitro*, we used chromogenic substrate method detection methods to measure DPP-IV. We found that isoquercitrin was a competitive inhibitor, with IC_50_ and *K*_i_ values of 96.8 and 236 μM, respectively. Isoquercitrin and sitagliptin also stimulated GLP-1 release in NCI-H716 cells. *In vivo*, a type 2 diabetic mouse model was established, and oral treatment with different concentration of isoquercitrin and sitagliptin for 8 weeks significantly decreased the fasting blood glucose level. The weight and the levels of serum GLP-1 and insulin of the mice in the isoquercitrin group were higher than those in the model group (*P* < 0.001). An oral glucose tolerance test showed that the isoquercitrin significantly inhibited postprandial blood glucose changes in a dose-dependent manner. These findings demonstrated the hypoglycemic effects of isoquercitrin and indicated that isoquercitrin improved insulin sensitivity by targeting DPP-IV.

## Abstract 2

Glucagon-like peptide (GLP)-1 is a potent glucose-dependent insulinotropic gut hormone released from intestinal L cells.

## Graphical abstract

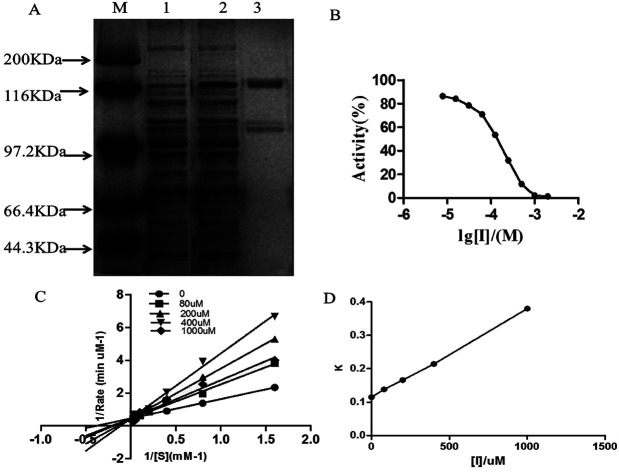

## Introduction

Diabetes is a worldwide epidemic, with the World Health Organization estimating that more than 220 million people have diabetes worldwide, with greater than 90% of those having type 2 diabetes mellitus (T2DM). T2DM is thought to develop as a combination of insulin resistance and pancreatic β-cell failure.^1^ Therefore, identification of novel treatments that would increase pancreatic insulin secretion while protecting pancreatic β-cells is of great interest. Incretin hormones, such as glucagon-like peptide-1 (GLP-1), are secreted from cells in the gastrointestinal (GI) tract into the circulation in response to nutrient absorption. They are a major component of the mechanism regulating post-prandial insulin secretion when insulin is needed following meals.^2^ However, GLP-1 is rapidly inactivated *in vivo* by circulating peptidases, mainly dipeptidyl peptidase 4 (DPP-IV), so that they cannot be used for therapeutic purposes. DPP-IV inhibitors provide clinical benefits in patients with T2DM by increasing the levels of glucose-lowering incretin hormones, such as GLP-1. Therefore, the aim of this analysis was to comprehensively assess the pre-clinical efficacy of DPP-IV inhibitors in Chinese T2DM patients and to evaluate whether the response to treatment varies with different types of DPP-IV inhibitors in such patients.^3^

Studies found that flavonoids have an obvious function in modulate DPP4 activity, such as grape seed-derived procyanidins and *Pilea microphylla*.^4,5^ Isoquercitrin is one of the major bioactive constituents of the apocynum and folium mori, and it widely exists in medicinal plants, food and beverage, fruits and vegetables. Isoquercitrin was shown to be the pharmacologically effective component with potent antioxidant, anti-inflammatory, antidepressant, antihypertensive, and lipid-lowering activities.^6,7^ In this study, a possible hyperglycaemic mechanism was investigated through an *in vitro* enzymatic reaction kinetics experiment. We also explored the strength of the hypoglycemic effect and attempted to better understand its mechanisms using cell experiments and a T2DM mouse model.

## Materials and methods

Recombinant *E. coli* containing the ΔDPP-IV plasmid was purchased from Beijing Yiqiaoshenzhou Biotechnology Company. A GLP-1 ELISA Kit and an insulin ELISA Kit were purchased from RD Company. All types of restriction endonucleases, gel extraction kits, Plasmid Miniprep kits and T4 DNA ligases were purchased from Fermentas. Triton-100, PEP and BZM were purchased from Sigma-Aldrich. Gly-Pro-PNA was purchased from Roche. NCI-H716 cells were purchased from ATCC America, and all cell culture reagents were from Hyclone. Mice with body weights of approximately 20 ± 2 g were purchased from the experimental animal center of Jilin University; isoquercitrin and sitagliptin were purchased from Aladdin (Xi'an, People's Republic of China). The kits for the analysis of total triglyceride (TG) and total cholesterol (TC) levels were purchased from Beijing BHKT. STZ, Tris and EDTA were purchased from Beijing DingGuo Biotechnology Company. A Blood Glucose Kit was purchased from Takala.

### Preparation of ΔDPP-IV

The cDNA was obtained with the PMD18 template, which encodes the catalytic domain of DPP-IV (ΔDPP-IV); synthesis was performed with polyadenylated mRNA, and the recombinant plasmid was transformed into *E. coli* Rosstta DE3 plysS and induced by IPTG. The catalytic domain of DPP-IV was amplified by PCR, and then the truncated enzyme was purified by a GST Resin column.

### ΔDPP-IV inhibition assays

The DPP-IV activities were measured by the addition of 10 μL of 10 mmol L^−1^ Gly-Pro-PNA (the substrate) to a buffer solution (pH 8.0) containing 0.1 mol L^−1^ NaCl, 1 mmol L^−1^ ethylene diamine tetra-acetic acid (EDTA), 10 μL and 50 ng DPP-IV and recombinant DPP-IV, respectively, along with or without different concentrations of isoquercitrin diluted in dimethyl sulfoxide (DMSO). After incubation for 10 min at 37 °C, the reactions were terminated with 0.1 mol L^−1^ NaHCO_3_, and the amount of the product, pentose nucleic acid (PNA), was measured by UV absorbance at a wavelength of 405 nm. The inhibitory potency of inhibitors was evaluated by IC_50_ values.

### Determination of inhibition kinetics

Substrate (Gly-Pro-PNA) at different concentrations was added separately to the reaction mixtures containing various concentrations of isoquercitrin. The absorbance at 405 nm was measured to determine the amount of PNA produced. The inhibition kinetic analysis was carried out using the Line weaver–Burk plot of 1/*v versus* 1/[S].

### Cell culture and stimulation

NCI-H716 cells were cultured in 1640 medium supplemented with 10% foetal bovine serum (FBS) at 37 °C in a humidified atmosphere with 5% CO_2_. NCI-H716 cells were incubated with various concentrations of isoquercitrin (dissolved in DMSO) for 6 h, 24 h, 48 h and 72 h. Sitagliptin was used in the positive control group. Our pre-experiments involved the use of a series of sitagliptin concentrations (10 μmol L^−1^, 50 μmol L^−1^ and 100 μmol L^−1^) for 6 h, 24 h, 48 h and 72 h. For the GLP-1 detection, cells were evaluated using a GLP-1 ELISA kit; the UV absorbance was then measured at a wavelength of 450 nm.

### Establishment of type 2 diabetic mouse model and treatment protocol

Ninety male Chinese Kunming mice, which were 8 weeks old and weighed 18–22 g, were used in the studies. All animal trial procedures instituted by the Ethical Committee for the Experimental Use of Animals and for Drug Safety Evaluation were followed. All 90 mice were housed five to a cage in a 12 : 12 h light/dark cycle at an ambient temperature of 22 °C-25 °C. For the normal group, 10 mice were fed ordinary chow, and the other 80 mice were fed a high-fat diet (composed of 20% sucrose, 10% pork lard, 2.5% cholesterol, 1% sodium cholate, and 66.5% ordinary chow) to induce type 2 diabetes. After 4 weeks, 80 mice were fasted for 8 h with free access to water and then intraperitoneally injected with STZ (35 mg kg^−1^ in 0.1 mol L^−1^ citrate-buffered saline, pH 4.4; injection for 3 days, and one injection per day) to induce type 2 diabetes. The STZ-treated mice had free access to high-fat food and water. After 1 week, the STZ-treated mice had a fasting blood glucose (FBG) level of 11.1 mmol L^−1^. These type 2 diabetic mice were separated into five groups (sixteen mice per group), including a diabetes model group that received 0.9% saline [vehicle], an isoquercitrin low-dose group that received 20 mg kg^−1^, isoquercitrin, a medium-dose group that received 40 mg kg^−1^, and an isoquercitrin high-dose group that received a 80 mg kg^−1^ dose of isoquercitrin (isoquercitrin was dissolved in dimethyl sulfoxide [DMSO] solvent; the sitagliptin group received 20 mg kg^−1^ doses of sitagliptin). The normal group received 0.9% saline (vehicle). All the drug stock solutions were diluted in 0.9% saline and administered through oral gavage once per day for 8 weeks.

### Measurement of fasting blood glucose, oral glucose tolerance test (OGTT) results, body weight, cholestenone and lipid profile

During the treatment period, the body weight and blood glucose of 8 h-fasted mice were measured every week. FBG was measured using a One Touch Ultra Easy glucose reader. Blood samples were obtained from the tail vein of mice. The total TC and TG in serum were measured following the recommended instructions in the commercial kits.

### Measurement of GLP-1 and insulin

After the treatment period, the mice were fasted for 8 h with free access to water and then received 2 g kg^−1^ doses of glucose. Blood samples were obtained from the tail vein of mice at different times (10 min, 20 min, 30 min, 1 h and 2 h). The samples were held for 30 min and then centrifuged at 5000 × *g* for 10 min at 4 °C. Afterward, the supernatant of each sample was collected. The levels of GLP-1 and insulin were measured using GLP-1 and insulin ELISA kits, respectively.

### Statistical analysis

Data are presented as the mean ± SD. Statistical analysis was conducted using Student's *t*-test or one-way ANOVA with GraphPad Prism 5 software. A probability value of *P* < 0.05 was considered statistically significant.

## Results

### Expression and purification of DPP-IV

**Fig. 1:**
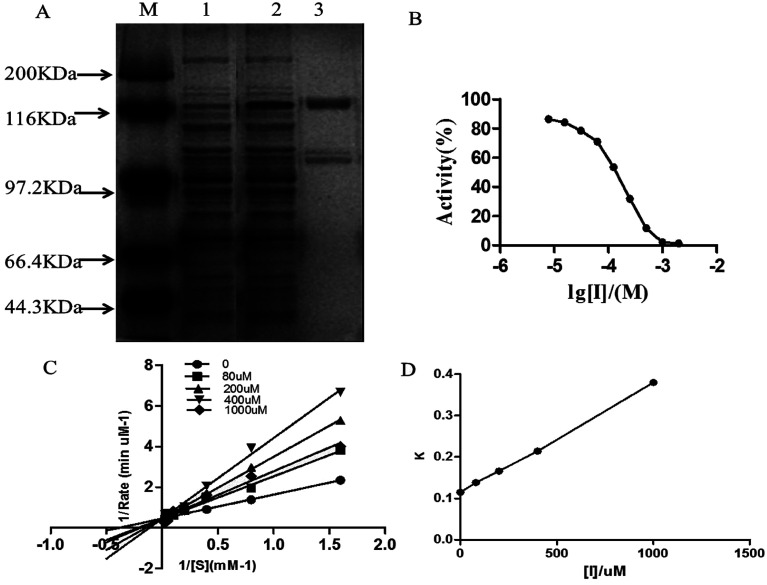
DPP-IV (A) expression and purification of DPP-IV. (lane A: marker; lane 1: crude extraction; lane 2: after induction; lane 3: after purification.) (B) IC_50_ value of isoquercitrin against DPP-IV; (C) inhibition type of isoquercitrin against ΔDPP-IV; (D) inhibition constant of isoquercitrin against ΔDPP-IV.

The expression of purified DPP-IV is shown in Fig. 1A. The specific activity was approximately 19 500 units per mg. Analysis of the SDS-polyacrylamide gel electrophoresis grey level indicated that the purity of DPP-IV was over 90%, which met the standard for inhibitor screening. The following experiments were then performed with purified DPP-IV.

### Exhibited potent DPP-IV inhibitory activity

Isoquercitrin had an obvious inhibition effect on DPP-IV from 50 types of monomer compounds. The inhibitory potency of isoquercitrin against DPP-IV was evaluated using the concentration-dependent inhibition curves, as shown in Fig. 1B. Isoquercitrin was identified as a DPP-IV inhibitor, with an IC_50_ of 96.8 μmol L^−1^. To further determine the inhibition type of isoquercitrin against DPP-IV, Lineweaver–Burk analysis was performed. The Lineweaver–Burk plot had a common intercept for five lines on the 1/*v* axis as the isoquercitrin concentration increased from 0 to 1000 μmol L^−1^, indicating that isoquercitrin inhibits DPP-IV by competing with the substrate for the enzyme active site (Fig. 1C). The inhibition constant (*K*_i_) was the inhibition constant of isoquercitrin against DPP-IV, and *K*_i_ was determined from the intercept of the *x*-axis; thus, *K*_i_ was calculated to be 236 μmol L^−1^ (Fig. 1D).

### Effects of isoquercitrin on GLP-1 secretion in NCI-H716 cells

**Fig. 2:**
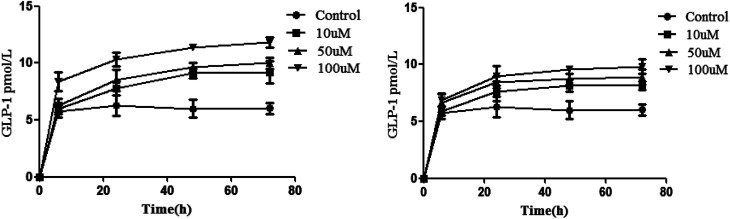
GLP-1 secretion in NCI-H716 cells. (Isoquercitrin; sitagliptin).

Inoculation was performed with 5 × 10^4^ NCI-H716 cells. We then measured the cell quantity and GLP-1 secretion every 24 h. The results showed that the level of GLP-1 secretion significantly increased after the cell density reached 2 × 10^5^/mL, so isoquercitrin and sitagliptin stimulated the cells when the density was 2 × 10^5^/mL. It was found that isoquercitrin treatment may increase GLP-1 secretion in a concentration-dependent manner (Fig. 2A), as determined using ELISA, and the level of GLP-1 significantly increases beginning at 6 h (*P* < 0.01), when the GLP-1 level increased from the basal level of 5.73 pmol L^−1^ protein to 8.37 pmol L^−1^ protein.

Furthermore, we also evaluated the GLP-1 release in a positive control. In this study, we employed the well-known sitagliptin as a positive control. The results indicated that sitagliptin dose-dependently increased the level of GLP-1 (Fig. 2B).

### Isoquercitrin decreases blood glucose levels and ameliorates body weight disorder in T2DM mice

Isoquercitrin affects the appearance and ameliorates the body weight disorder in T2DM mice. At the beginning of the study, we established a T2DM mouse model by high-fat feeding and treatment with low-dose STZ. The FBG of mice reached 11.1 mmol L^−1^, whereas the TG had no significant change (Fig. 4C and D). These results confirmed the successful establishment of the T2DM model. It was very obvious that the model mice with polydipsia and polyuria lost body weight; the water consumption in the model group was greater than that in the normal group, the hair was extremely dull, and the spirit was listless. The condition of the isoquercitrin group was better than that of the model group.

**Fig. 3:**
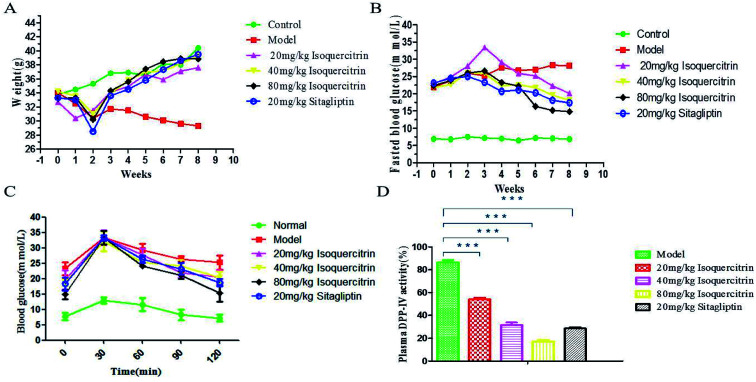
Effect of isoquercitrin on body weight, blood glucose and DPP-IV activity in T2DM mice. ((A) effects of drugs on the body weight; (B) effects of drugs on blood glucose levels; (C) OGTT results; (D) plasma ΔDPP-IV activity of mice.*** *P* < 0.001; ** *P* < 0.01; * *P* < 0.05).

The body weight stably increased in the normal mice but decreased in the model mice (Fig. 3A). The value in the isoquercitrin-treated mice and sitagliptin-treated mice decreased during the early treatment and then gradually increased, and there were not an obvious difference compared to the normal group. The weight of the mice in the isoquercitrin high dose group and sitagliptin group were higher than that in the low dose group, and the change occurred in a dose-dependent manner.

The body weight significant decreased in diabetic model mice after 8 weeks, whereas the weight increased in the other groups of mice. The body weight of the mice in the isoquercitrin group and the sitagliptin group were higher than that before treatment (*P* < 0.001). The body weight exhibited no statistically significant difference at the end of treatment in the isoquercitrin-treated mice compared with normal mice and sitagliptin-treated mice. These results suggest that isoquercitrin can ameliorate body weight disorder in T2DM mice.

### Isoquercitrin decreases the blood glucose levels and glucose tolerance of T2DM mice

We administered isoquercitrin, sitagliptin or vehicle for 8 weeks to these mice. The FBG in the diabetic model mice gradually increased and was significantly higher than that in the normal mice (Fig. 3B). The blood glucose level increased significantly in the diabetic model mice after 8 weeks. At the end of the 8 weeks of treatment, the blood glucose level in the high dose isoquercitrin-treated mice and the sitagliptin group was significantly decreased compared with that in the diabetic mice (*P* < 0.001). However the blood glucose levels of the low-dose group and medium-dose group mice declined slightly. These results indicated that isoquercitrin was an efficient hypoglycemic reagent, although further *in vivo* experiments, particularly with various dosages, are required to better estimate its therapeutic potential. A OGTT showed that the blood glucose concentration significantly decreased in the treatment group compared with that in the model group. The medium-dose and high-dose isoquercitrin treatment and sitagliptin obviously antagonized the blood glucose rise caused by exogenous glucose, and low doses of isoquercitrin also reduced the blood glucose level (Fig. 3C).

### Effects of isoquercitrin treatment on the DPP-IV activity, GLP-1 level and insulin level in serum

To address how isoquercitrin exerts its effect on the amelioration of hyperglycaemia in T2DM mice, we investigated the DPP-IV activity and GLP-1 secretion levels in the serum. The DPP-IV activity in serum rapidly decreased in the isoquercitrin- and sitagliptin-treated diabetic mice. Different concentrations of isoquercitrin have an obvious inhibitory effect in the serum of DPP-IV in mice treated for 8 weeks, which showed the obvious dose-dependent increase in the inhibitory effect of DPP-IV (Fig. 3D).

**Fig. 4:**
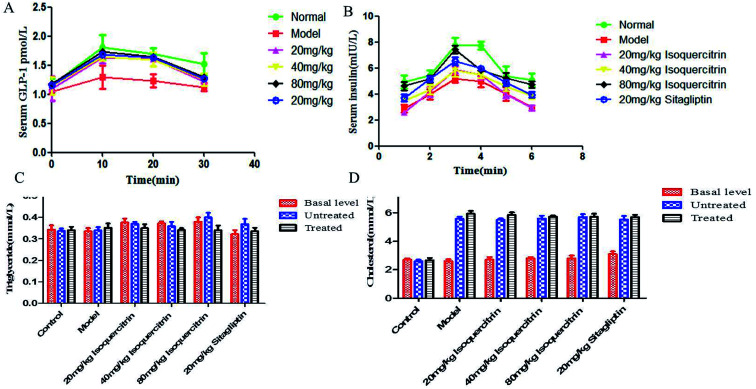
Effect of isoquercitrin on GLP-1, insulin, TC and TG levels in T2DM mice. (A) Effects of drugs on GLP-1; (B) effects of drugs on the insulin level at different times; (C) triglyceride; (D) cholesterol level (B).

The GLP-1 levels in portal plasma were investigated after a glucose load (Fig. 4A), and the area under concentration–time curves from 0 to 30 min was estimated using the linear trapezoidal rule. It was found that a glucose load might induce GLP-1 release. The serum levels of GLP-1 of the mice in the isoquercitrin group and the sitagliptin group were higher than those in the model group (*P* < 0.001). The high dose of isoquercitrin (80 mg kg^−1^) may enhance the GLP-1 release induced by the glucose load, and the peak level occurred at 10 min. The GLP-1 level rose from 1.04 to 1.3 pmol L^−1^ in model group mice and increased obviously in the high dose group mice, with the level increasing from 1.15 to 1.72 pmol L^−1^ (*N* = 10). The level of serum GLP-1 was decreased in all of groups at 20 min. However, the level almost decreased to the original level after 30 min. The GLP-1 level of the mice in model group increased slightly after glucose stimulation, which may result from the high DPP-IV activity *in vivo*, and GLP-1 was degraded.

### Effects of isoquercitrin treatment on insulin levels

Plasma insulin levels were measured following a glucose load (Fig. 4B). It was found that a glucose load might induce an increase in the insulin level in plasma, and the peak concentration occurred at 20 min. The plasma insulin level significantly increased in a dose-dependent manner, with the value increasing from 4.6 mIU L^−1^ to 7.4 mIU L^−1^ in the high-dose isoquercitrin group (*N* = 10). The level of insulin decreased in all groups at 30 min. However, the level almost decreased to the original level after 120 min. The levels of serum insulin in the mice of the isoquercitrin group and the sitagliptin group were higher than those in the model group at the same time, especially in the high-dose isoquercitrin group (*P* < 0.001). Although treatment with the low dose of isoquercitrin tended to decrease the insulin level, the change was not significant.

### TC and TG characteristics of the lipid profile in the serum

The TC in serum was significantly higher after T2DM inducement than before inducement, whereas TG did not increase. The pre- and post-treatment TC did not differ upon isoquercitrin or sitagliptin treatment (Fig. 4C). The TG in serum was lower after treatment than before treatment, but the difference was not significant (Fig. 4D).

## Discussion

T2DM is characterized by increased levels of blood glucose due to impaired insulin sensitivity (insulin resistance). This study revealed several essential findings relevant to the hypoglycemic effect of isoquercitrin and proposed a new underlying mechanism, independent of the findings in previous studies. Therefore, the advised inhibition of DPP-IV activity is considered to evoke multiple sets of physiological responses to resist T2DM and obesity *via* facilitating tyrosine phosphorylation of insulin signalling molecules. In fact, several studies have reported that oral administration of a DPP-IV inhibitor can decrease glucose levels in T2DM mice.^8–11^

Pharmacological approaches for treating T2DM and obesity have focused partly on targeting the glucagon-like peptide-1 (GLP-1) system.^12–15^ GLP-1 is an incretin hormone produced in the L cells of the intestine that acts at the GLP-1 receptor (GLP-1R) to improve glycaemic control, reduce food intake, and improve insulin sensitivity.^16^ Studies demonstrated that the role of GLP-1 in stimulating insulin secretion is significantly reduced in type 2 diabetes, which has been attributed to a decrease in GLP-1 receptor expression.^17^ Because native GLP-1 is rapidly degraded by the enzyme dipeptidyl peptidase-IV (DPP-IV),^18,19^ pharmacological strategies include inhibiting DPP-IV activity and creating a GLP-1R agonist resistant to enzymatic degradation. Thus, DPP-IV could be one of the first candidates for the target molecule of isoquercitrin through which isoquercitrin acts to improve insulin sensitivity. As our findings indicated, isoquercitrin competitively inhibited DPP-IV activity *in vitro* by directly blocking the DPP-IV active-site pocket.

NCI-H716 cells served as *in vitro* models of intestinal L cell in further investigations of the effect of isoquercitrin in regulating GLP-1 release. Reports indicated that L cells secrete GLP-1 in response to stimulating the intestines, the concentration of GLP-1 depends on the islet beta cell expression of the GLP-1 receptor, and insulin secretion reduced the production of liver glycogen.^20^ A study on the hypoglycemic effect of berberine also found that berberine decreased blood glucose by increasing the secretion of GLP-1.^21^ An *in vitro* study demonstrated that isoquercitrin may stimulate GLP-1 secretion from NCI-H716 cells in a dose-dependent manner; this change was accompanied by an increased GLP-1 level, which indicated that isoquercitrin may increase GLP-1 release in addition to promoting GLP-1 biosynthesis.

It was obvious that treatment through oral gavage with different concentrations of isoquercitrin resulted in a profound attenuation in hyperglycaemia. In particular, the strength of this hypoglycemic effect was comparable to that of sitagliptin (clinical drug). The anti-hyperglycaemic effect of sitagliptin, an antidiabetic drug, is due to the suppression of DPP-IV activity, which is same mechanism as for isoquercitrin, as we demonstrated. However, our findings in this study are the first, to the best of our knowledge, to demonstrate that isoquercitrin inhibits DPP-IV *in vitro* and *in vivo*, resulting in enhanced insulin secretion.

For type 2 diabetic patients, hyperglycaemia is often accompanied by dyslipidemia, which manifests as abnormal serum lipid profiles, such as TG and TC changes. Abnormal values of these indices result in a series of metabolic disorders and complications. This study indicated that the effect of isoquercitrin on serum TC and TG for the studied mice was not statistically significant. However, we found that isoquercitrin can alleviate bodyweight disorders, implying some multi-target mechanisms remain to be determined.

Here, we demonstrated that oral treatment with isoquercitrin resulted in a profound attenuation in hyperglycaemia in diabetic mice. The mechanism may be associated with the DPP-IV activity inhibition by isoquercitrin, but more studies are needed. An inhibitor of DPP-IV could stimulate the secretion of insulin by improving the activity, which significantly improved symptoms in type 2 diabetes.^22^ The level of GLP-1 in plasma is associated with the activity of DPP-IV. A study showed that DA-1229, an inhibitor of DPP-IV, had an obvious inhibitory effect *in vivo*. Treatment with 300 mg kg^−1^ day^−1^ DA-1229 through oral gavage resulted in an obvious inhibitory effect in the mouse serum, which is similar to our results.^23^ Studies suggested that different quercetin reduced the blood sugar in type 2 diabetes mice by inhibiting α-glycosidase enzyme activity, with the effect depending on whether the tested dose was 50 mg kg^−1^ or 200 mg kg^−1^.^24^ However, our findings in this study are the first, to the best of our knowledge, to demonstrate that isoquercitrin inhibits DPP-IV *in vitro* and *in vivo*, resulting in enhanced GLP-1 and insulin secretion and then amelioration of hyperglycaemia in high-fat diet and STZ-induced T2DM mice.

*In vivo* studies showed that isoquercitrin treatment might lower the fasting blood glucose level, and this change is accompanied by an increase of insulin levels in plasma. Treatment with 80 mg kg^−1^ isoquercitrin might significantly increase the GLP-1 release induced by a glucose load, with this change accompanied by a higher GLP-1 level in portal plasma. Peripherally released GLP-1 enters brain areas and participates in the regulation of the anorexic response,^25^ which indicates that the suppression of food intake induced by isoquercitrin might partly lead to the enhancement in GLP-1 release. It is well-known that GLP-1 exerts important effects on regulating glucose homeostasis *via* stimulating insulin secretion, beta-cell proliferation, inhibiting food intake.^26^ All of these results further supported our previous findings that isoquercitrin exerted its antidiabetic effects partly *via* increasing GLP-1 release. These findings further indicated the possibility of enhancement of GLP-1 biosynthesis by isoquercitrin.^27,28^ A study showed that the insulin secretion stimulated by GLP-1 *in vivo* was consistent with that found in the OGTT, which was consistent with our experimental results.

Although we have proved the hyperglycaemic effects of isoquercitrin on the type 2 diabetes mice occur by targeting DPP-IV, there are also some limitations for this study. First, there are many other pathways affecting type 2 diabetes other than the inhibition of DPP-IV. Second, there may be several other substrates of DPP-IV in cells, which may have some adverse effects on the complete or partial inhibition of DPP-IV. Therefore, if applying isoquercitrin into clinical therapy, the side effects of isoquercitrin must be investigated in future studies.

## Conflicts of interest

The authors declare that there is no conflict of interests regarding the publication of this paper.

## Supplementary Material
